# Description of a Culture-Sensitive, Low-Threshold Psychoeducation Intervention for Asylum Seekers (Tea Garden)

**DOI:** 10.32872/cpe.4577

**Published:** 2021-11-23

**Authors:** Ricarda Mewes, Julia Giesebrecht, Cornelia Weise, Freyja Grupp

**Affiliations:** 1 Outpatient Unit for Research, Teaching and Practice, Faculty of Psychology, University of Vienna, Vienna, Austria; 2 Division of Clinical Psychology and Psychotherapy, Department of Psychology, University of Marburg, Marburg, Germany; University of Lausanne, Lausanne, Switzerland

**Keywords:** asylum seekers, culture-sensitive, knowledge, mental health (care), psychoeducation, transdiagnostic

## Abstract

**Background:**

Asylum seekers often suffer from high levels of mental distress. However, as a result of a lack of knowledge about mental health and health care, as well as cultural and language barriers, the utilization of mental health care in Western host countries is often difficult for these individuals. Reducing these barriers may thus be a crucial first step towards appropriate mental health care. Previous research showed that psychoeducation may be helpful in this regard.

**Method:**

The current manuscript describes a short, low-threshold and transdiagnostic intervention named ‘Tea Garden (TG)’. The TG aims to increase specific knowledge about mental health problems and available treatments, and may improve psychological resilience and self-care. In this manuscript, we specifically focus on culturally sensitive facets, following the framework proposed by Heim and colleagues (2021, https://doi.org/10.32872/cpe.6351), and lessons learned from three independent pilot evaluations (Ns = 31; 61; 20).

**Results:**

The TG was found to be feasible and quantitative results showed that it was helpful for male and female asylum seekers from different countries of origin (e.g., Afghanistan, Syria, Pakistan, Iraq) and with different educational levels. Interestingly, even asylum seekers who had already been in Germany or Austria for three or more years benefited from the TG.

**Conclusion:**

The TG specifically aims to be culture-sensitive rather than culture-specific, to be transdiagnostic rather than focused on specific mental disorders, and to be suitable for asylum seekers who are still in the insecure process of applying for asylum. It may also be helpful for distressed asylum seekers who do not fulfill the criteria for a mental disorder, and for healthy asylum seekers who could use the knowledge gained in the TG to help others.

## Highlights

- There is a lack of short, low-threshold, and culture-sensitive interventions for asylum seekers.
- A transdiagnostic intervention, named ‘Tea Garden’ (TG), is described and findings of pilot evaluations are reported.
- The TG aims to increase knowledge about mental health (care), and improve resilience and self-care.
- The TG was found to be helpful for refugees from different origins and with different educational levels.

The prevalence of mental disorders in refugees and asylum seekers^1^1'An asylum seeker' is defined as a person who is seeking international protection and whose claim for asylum has not yet been finalized. If protection is granted according to the 1951 Refugee Convention, the person is recognized as a 'refugee'. Accordingly, all refugees were initially asylum seekers. Many of the studies cited investigated both asylum seekers and refugees. Because of the current study's focus, we primarily use the term 'asylum seekers' and speak of refugees only if they are specifically addressed. is high, as they have frequently experienced different kinds of hardship and traumatic situations (Blackmore et al., 2020; Henkelmann et al., 2020). Even when asylum seekers have arrived in a safe host country, factors such as a lengthy asylum procedure, fear of deportation, or ethnic discrimination pose a risk for the aggravation or new manifestation of mental health problems (Gleeson et al., 2020). Despite high levels of mental distress, access to mental health treatment for asylum seekers is limited (Björkenstam et al., 2020; Führer et al., 2020). This is mainly a result of barriers such as a general lack of knowledge about mental disorders and mental health care, but is also caused by limited access to health care systems in host countries, cultural understanding and stigmatization of mental disorders and mental health care, and language barriers (Grupp et al., 2019; Mårtensson et al., 2020).

Systematic reviews have revealed that psychoeducation improves specific knowledge about mental disorders (e.g., about possible causes, typical symptoms of a disorder, factors influencing the symptoms) and psychosocial functioning, including coping with symptoms, and reduces distress for people suffering from mental disorders (Barnicot et al., 2020; Tursi et al., 2013). Colom (2011, p. 339) defines psychoeducation “as a patient’s empowering training targeted at promoting awareness and proactivity, providing tools to manage, cope and live with a chronic condition …, and changing behaviours and attitudes related to the condition.” Consequently, psychoeducation may be an effective intervention to improve the mental health knowledge and to enable an initial mental health improvement of different cultural groups of asylum seekers in Western host countries. Such a psychoeducation intervention should be adapted to a degree that allows its use in different cultural groups (i.e., being culture-sensitive; e.g., see the suggestions on cultural adapted cognitive behavioral therapy by Hinton et al., 2012) rather than focused on one specific culture or group (i.e., culture-specific; e.g., see the examples and recommendations for specific cultural/ethnic groups by Smith et al., 2011). However, this type of psychoeducation for asylum seekers is lacking. Therefore, we developed a short, culture-sensitive intervention, named Tea Garden (TG). We chose this name because a tea garden is a familiar concept for many migrants from different regions of origin and is often associated with a positive situation. We wanted to avoid difficult names or labels (e.g. psychotherapy or psychological) that could discourage interested persons as a result of a lack of knowledge about psychotherapy and psychological interventions or a possible fear of stigmatization. Our focus was on reducing distress and increasing knowledge about the development of mental disorders, resources to cope with mental distress, and interventions, that were assumed to be of particular relevance for asylum seekers. In line with Betsch and colleagues, we aimed for a “deliberate and evidence-informed adaptation of health communication to the recipients’ cultural background in order to increase knowledge and improve preparation for medical decision making and to enhance the persuasiveness of messages in health promotion” (Betsch et al., 2016, p. 813). We did not limit culture to the nationality or a set of habits and beliefs, but also account for the particular sociodemographic, legal, and living situation of asylum seekers (e.g., Napier et al., 2014).

Our main aim (I) in this paper is to describe the TG with a specific focus on culturally sensitive facets, following the framework proposed by Heim and colleagues (2021, this issue). Additionally, we provide summarized findings (II) from pilot evaluation studies with regard to the acceptability and feasibility of the TG, as well as lessons learned.

## Description of the Tea Garden (TG)

The TG was developed as part of the project ‘Psychotherapeutic first aid for asylum seekers living in Hesse’ funded by the European Refugee Fund, EFF-12-775 (Mewes et al., 2015). The aim of the TG is threefold: (1) to increase knowledge about mental disorders most relevant for asylum seekers, psychological and psychiatric treatments, mental health care in the resettlement country, and the special access conditions for asylum seekers in this regard; (2) to reduce stigmatization of mental disorders and mental health care, and thereby increase openness to psychotherapy and psychiatric treatments; and (3) to strengthen psychological resources and achieve first reduction of mental distress.

### Team of Developers

The developing team comprised members from different countries of origin and different cultural backgrounds (e.g., Persian, Arabic, Kurdish, Turkish), some of them with a refugee background, psychotherapists working with asylum seekers, and researchers in the field of intercultural psychology.

### Target Population

The target group for the TG consists of asylum seekers who have recently arrived in a host country (e.g., max. 18 months), are still in the process of applying for asylum, and may suddenly be transferred to other cities or Federal states during their asylum procedure. Participants may be mentally distressed or suffer from a mental disorder, but this is not mandatory for participation. The TG is transdiagnostic and may even be helpful for healthy asylum seekers who could use the knowledge gained in the TG to help others.

### General Implementation of the TG

With the aid of interpreters, the TG is provided in a group format to provide help to several asylum seekers simultaneously. The TG consists of four modules (A-D): Module A) establishing trust and confidence; Module B) symptoms of mental disorders; Module C) resources and self-care, and Module D) treatment options. These modules are interactively presented in two 90-minute sessions delivered one week apart in groups of approximately six participants (detailed information can be found in the German manual, Mewes et al., 2015). This schedule is considered short enough to reach many target clients, but long enough to provide the required information in a relaxing and interactive manner.

A group setting is applied to enhance social support and mutual exchange, and to take into account the mainly collectivistic background of the main groups of asylum seekers (in Western host countries) as well as shared pre-, peri-, and postmigration experiences (Kananian et al., 2017; Kira et al., 2012). These benefits are assumed to outweigh possible disadvantages, such as reservations to participate in a group (e.g., worries about confidentiality and being stigmatized), the limited consideration of individual problems, and the therapists’ necessity to closely monitor not only the content but also the group process (Kira et al., 2012).

Tea and food are offered to promote a relaxing and welcoming atmosphere. In addition, the TG uses images/ illustrations, symbols (e.g., rope, flowers, stones, spinning top) and familiar metaphors in order to facilitate communication and to adapt to different educational levels. Its material is free of written language or complicated figures, and operates best in gender- and language-homogenous groups of five to seven participants.

### Components and Contents of the Intervention

Based on a literature review and our own work (Hinton et al., 2012; Reich et al., 2015) as well as advice from experienced psychotherapists in the field, we included several treatment components in order to foster confidence and therapy motivation, and thus to increase the usefulness of the TG. With regard to *specific components,* i.e. components that have specific relevance for the aims of the TG, we focused on psychoeducation (e.g., explaining that traumatic events can cause symptoms, explaining the concept of psychotherapy), strengthening resources (e.g., introducing possible resources, initiating exchange about useful strategies for coping and how to implement them in the daily life), giving hope (e.g., by explaining that symptoms can improve with the right care), and reducing stigmatization (e.g., by initiating exchange about problems and by emphasizing that persons with mental problems are not ‘mad’). In addition, we included several *unspecific components* that should support the implementation of the TG (but do not specifically relate to the aims of the TG) such as guiding through the sessions (e.g., by outlining the structure of the sessions and monitoring the time), normalizing (e.g., by explaining that experiencing symptoms such as worries and flashbacks after traumatic events is normal), discussing advantages of and barriers to treatments (e.g., by asking for the participants’ views on psychopharmacological treatments, by explaining how to get a psychotherapy and addressing possible barriers), monitoring the distress level of participants (e.g., by working with two therapists and a limited number of participants, one therapist can watch out for signs of distress), and interrupting participants when narratives become too personal/ distress becomes too high (this is part of a set of group rules which are introduced at the first session). Moreover, *in-session techniques* such as behavioral experiments (relaxation) and exchange between group participants were included to this end.

In order to consider relevant *target syndromes, needs, and concepts of distress* (Lewis-Fernández & Kirmayer, 2019) of our target group, the following contents were included in the TG:

- *Explanatory models, etiological assumptions*. Based on a literature review (e.g., Liedl et al., 2010), we used a body-mind metaphor for the description of a traumatic event and the care and healing related to this event (i.e., the mind can be wounded by traumatic events; this wound is similar to a wound on the hand after a cut; wounds in the mind may cause symptoms; and the wound must be nursed and will then heal, leaving a scar).
- *Symptom patterns and socially acceptable terms for expressing distress.* The higher relevance of bodily symptoms in many groups of immigrants in Western host countries and culture-specific symptoms such as 'burning liver' or 'pulling hair' was accounted for by explicitly introducing these symptoms (among others) with drawings as part of a module about symptoms (Module B). This decision was based on a literature review (e.g., Hinton et al., 2012; Rometsch et al., 2020) and experiences from the team of developers.

*Culturally salient resources*. As many groups of asylum seekers in Western host countries highly value religion and faith, and have strong ties within the ‘extended family’, these potential resources were introduced as part of the module on resources and self-care (Module C). This decision was based on a literature review, our own scientific work (e.g., Grupp et al., 2019), and advice from the team of developers.

### Suggested Outcome Measure

In line with trials offering psychoeducation interventions for persons with serious mental illnesses (e.g., Zhao et al., 2015), the primary outcome for evaluations of the TG should be *changes in specific knowledge* with regard to mental health (please see the Appendix in the Supplementary Materials for suggestions on measures of the other aims of the TG). Moreover, the feasibility and acceptability of the TG should be assessed, e.g., the atmosphere, the comprehensibility, and the communication, as well as the personal benefit, relief, and perceptions of resources.

For the three pilot studies reported below, a questionnaire developed by our work group was used (Demir et al., 2016). This questionnaire assessed self-reported knowledge on 1) symptoms of mental disorders, 2) resilience and coping strategies, and 3) mental health care offered in the country. To facilitate assessment in illiterate and low-educated participants, we aimed for easy language and used smileys to indicate negative to positive response or low to high agreement and a right-angled triangle symbol to indicate increase in knowledge (range 1 = *not at all* to 5 = *very much*; the higher the value the more positive the assessment), respectively. Moreover, feedback on the personal benefits, and suggestions for improvement, could be given using free text.

## Findings From First Evaluations of the Tea Garden and Lessons Learned

Three independent pilot evaluations were conducted with a focus on acceptance, feasibility, first hints of possible effectiveness, as well as lessons learned (mainly based on anecdotal reports of the researchers, and the therapists who conducted the TGs, and written and verbal feedback of participants). Two pilot evaluations were conducted in Germany (Bogdanski et al., 2019; Demir et al., 2016) and one in Austria. Most participants came from Syria, Afghanistan, Pakistan, or Iraq. More detailed information is provided in the Appendix (see Supplementary Materials). By reason of the low-threshold character of the TG, participants in the pilot evaluations were not screened for mental disorders. The outcome assessments were conducted after each TG session and were supported by interpreters when necessary. After the TG, participants reported increased knowledge about mental health care, psychotherapy and self-help options, relief for general distress, improved perceptions of resources, and high overall satisfaction with the program.

**Lessons learned:**
- To facilitate recruitment, potential participants needed to be educated in detail about the program, and it was necessary to establish trust, be patient, and build a network of contact persons.
- The outcome assessment was too complex and unfamiliar for some participants, and was simplified by only using smileys.
- Some participants erroneously expected to learn about asylum procedures. Therefore, flyers and invitations should be phrased very clearly and highlight the content of the TG.
- Even asylum seekers with longer durations of stay (e.g. three years and more) appreciated the TG.
- The illustrations used in the TG were complemented by new illustrations in order to enhance the variety of shown human appearances and the fit for different groups of asylum seekers.
- The larger the size of the group the more likely conflicts between participants may emerge. We thus suggest to limit the number of participants to eight.

## Discussion

In contrast to other interventions, the TG specifically aims to be culture-sensitive rather than culture-specific, to be transdiagnostic rather than focusing on specific mental disorders, and to be suitable for asylum seekers who are still in the insecure process of applying for asylum. The three independent pilot evaluations demonstrated the feasibility of the TG and its acceptance with regard to different countries of origin, spoken languages, educational levels, and durations of stay in the host countries. Moreover, they provided us with important lessons for the future recruitment of potential participants, appropriate designs for the outcome assessment, the materials used, and the recommended group size. In general, our findings suggest that the TG may be a useful first step to improve mental health care for asylum seekers. However, the generalizability and explanatory power of the presented results is limited by the single-group designs, and the lack of pre-post comparisons as well as follow-up assessments that would provide information about the sustainability of possible benefits. These limitations will now be tackled by the multicenter randomized controlled trial ‘Efficacy of Low-threshold, Culturally Sensitive Group Psychoeducation in Asylum Seekers’ (LoPe; DRKS00020564), where the participants will be randomized to either the TG or a waitlist control group and changes in knowledge will be assessed pre- and postintervention as well as two and six months later.

Following the example of other projects that successfully used brief psychological interventions to reduce the treatment gap for common mental disorders in affected groups, such as the Friendship Bench project in Zimbabwe (Chibanda et al., 2016) or the Self-Help Plus project in Uganda (Tol et al., 2020), the TG might best be implemented via psychologists working in asylum facilities, trained and supervised social workers or even lay facilitators, depending on the local means and structures. By being culture-sensitive and very low-threshold, the TG considers the high diversity of asylum seekers living in Western host countries (e.g., with regard to their countries of origin, their ethnicity, religion, education level, asylum status, distress level, etc.) and avoids the discrimination of specific (often particularly marginalized) groups. The TG may, thus, be considered as a broadly applicable first-line mental health intervention.

## Supplementary Materials

The Supplementary Materials contain suggestions for the measures of the aims two and three of the Tea Garden and more detailed information about findings from the first evaluations of the Tea Garden and lessons learned (incl. two Tables) (for access see Index of Supplementary Materials below).

10.23668/psycharchives.5030 Supplement 1 Supplementary materials to "Description of a culture-sensitive, low-threshold psychoeducation intervention for asylum seekers (Tea Garden)"

MewesR.
GiesebrechtJ.
WeiseC.
GruppF.
 (2021). Supplementary Materials to "Description of a culture-sensitive, low-threshold psychoeducation intervention for asylum seekers (Tea Garden)"
[Additional information]. PsychOpen. 10.23668/psycharchives.5030
PMC967083036405677
