# Supplementary material for: Description of a Culture-Sensitive, Low-Threshold Psychoeducation Intervention for Asylum Seekers (Tea Garden)
Source: Clin Psychol Eur. 2021 Nov 23;3(Spec Issue):e4577. doi: 10.32872/cpe.4577 (PMC9670830; doi:10.32872/cpe.4577)
Supplement: Supplement 1 [file cpe-03-4577-s01.pdf]

Appendix to the manuscript:

Description of a culture-sensitive, low-threshold psychoeducation intervention for asylum seekers (Tea Garden)

Ricarda Mewes[1]*, Julia Giesebrecht[2]*, Cornelia Weise[2], Freyja Grupp[2]

*Shared first authorship

[1] Outpatient Unit for Research, Teaching and Practice, Faculty of Psychology, University of Vienna, Vienna, Austria.

[2] Division of Clinical Psychology and Psychotherapy, Department of Psychology, University of Marburg, Germany.

Manuscript published in *Clinical Psychology in Europe*
https://doi.org/10.32872/cpe.4577

Content

Suggestions for the assessment of the aims two and three of the Tea Garden 2

Findings from first evaluations of the Tea Garden and lessons learned 2

Table 1: Sociodemographic data of participants from all three pilot evaluations 6

Table 2: Mean scores of the third pilot evaluations and correlations to duration of stay 7

References 8

Suggestions for the measures of the aims two and three of the Tea Garden

To assess the effects of the Tea Garden (TG) with regard to its additional aims, i.e., (a) reducing stigmatization of mental disorders and mental health care and increasing openness to psychotherapy and psychiatric treatments; and (b) strengthening psychological resources and achieving first reduction of mental distress, additional questionnaires such as the Attitudes Toward Seeking Mental Health Services (IASMHS;(Mackenzie et al., 2004)), the Connor-Davidson Resilience Scale (CD-RISC;(Connor & Davidson, 2003)), and the General Health Questionnaire (GHQ-28;(Alhamad & Al-Faris, 1998; Goldberg, 1978; Ormel et al., 1989)) may be used as well as qualitative methods.

Findings from first evaluations of the Tea Garden and lessons learned

Below, we summarize the results of three independent pilot evaluations with regard to acceptance, feasibility, and first hints of possible effectiveness of the TG, as well as lessons learned (mainly based on anecdotal reports of the researchers, the therapists who conducted the TGs, and written and verbal feedback of participants). Please note that by reason of the low-threshold character of the TG, participants in the pilot evaluations were not screened for mental disorders.

1. The ***first pilot evaluation*** was conducted as part of the project ‘Psychotherapeutic first aid for asylum seekers in Hesse’ (EFF-12-775) at the University of Marburg, Germany (Demir et al., 2016). In this pilot study, 31 asylum seekers participated in the TG (for sociodemographic data see Table 1 below). At the time of participation, they had been in Germany for between one week and three

months. Assessments were conducted at the end of each session of the TG. After the TG (presented in Arabic and Kurdish), participants reported increased knowledge about mental health care, psychotherapy and self-help options, relief for general distress, improved perceptions of resources, and high overall satisfaction with the program. On a scale from 1 to 5 (with higher values indicating greater improvement; see ‘outcome assessment’ above), all mean values ranged between 3.5 (SD=1.4) and 4.8 (SD= 0.4).

Lessons learned from evaluation 1: The reports of the researchers showed that participants were often suspicious about revealing personal information, because they feared a negative influence on their asylum process. They were often unfamiliar with study procedures and skeptical about the benefit of participating. To facilitate recruitment, potential participants needed to be educated in detail about the program, and it was necessary to establish trust, be patient, and build a network of contact persons.

2. The ***second pilot evaluation*** was conducted by the Division of Psychotherapy and Systems Neuroscience, University of Giessen, Germany, in close cooperation with the local health authorities of the county Lahn-Dill, Hesse (Bogdanski et al., 2019). The TG was provided in Dari, Farsi, Arabic, and Urdu for participants from Afghanistan, Syria, Pakistan, Iraq, and Morocco (see Table 1). Participants had already been living in Germany for an average of 34 months. Eighteen participants completed the outcome assessment after the first session of the TG and 43 participants after the second session. The participants rated the TG and the exchange with the other participants as valuable, and felt relieved and safe during their participation. After participation, they reported increased knowledge about symptoms of mental disorders and mental health care, and were more confident about handling their symptoms in the

future. The duration of stay in Germany did not influence the results (ANOVAs; all ps >.05), but comparisons between men and women showed that women rated the second session higher than did men in terms of usefulness and knowledge about mental problems and their own resources (ANOVAs; ps <.05).

Lessons learned from evaluation 2:

i) The use of questions and triangles in the outcome assessment led to inquiries and confusion during the evaluation, as they were too complex and unfamiliar for some participants. Therefore, the outcome questionnaire was simplified by only using statements and smileys.

ii) There were some dropouts after the first session, because participants were disappointed that they did not learn anything about asylum procedures. To better inform participants about what to expect from the TG, flyers and invitations should be phrased very clearly and highlight the content of the TG.

iii) In contrast to the assumption that only recently arrived asylum seekers would be interested in the TG, even asylum seekers and refugees with longer durations of stay appreciated the TG and benefited from it.

3. The ***third pilot evaluation*** was conducted in the context of a *Caritas Austria* project called “MIT” (*m*obile *i*ntervention *t*eam) in 2018/2019. Clinical psychologists visited residential facilities of basic welfare support for refugees in Vienna to provide information about mental disorders and mental health care. The goal was to identify those people who needed psychotherapeutic treatment. The TG was applied in two groups of women and two groups of men, each assisted by an interpreter. Participants who evaluated the TG came from Iraq, Afghanistan, Syria, and Iran. 65% of the

participants had been in Austria for 3.5 years (20% for around one year and 15% for 4.5 years) and 75% were asylum seekers (25% had been granted asylum). All mean values of the outcome questionnaire ranged from 3.1 (SD=0.9) to 4.4 (SD=0.9; See Table 2, below); knowledge about treatments was rated the lowest (probably due to the lack of module D for some participants) and comprehensibility the highest. No associations were found between the duration of stay and the outcome measures (r_s: all ps.>.05; Table 2). According to the written feedback, participants were very grateful and valued being reminded of self-help strategies, feeling safe, comfortable, relieved and self-empowered, growing knowledge about psychologists and treatment options and being able to remember information. After the TG, two participants felt enabled to seek psychotherapy.

Lessons learned from evaluation 3:

i) The illustrations used in the TG up to that time point had to be complemented by new illustrations in order to enhance the variety of shown human appearances and the fit for different groups of asylum seekers and refugees.

ii) The larger the size of the group (one group was conducted with 17 participants from one facility), the more likely it may be that residents who are in conflict with each other will be participating in the same group. This can compromise the group atmosphere and result in an unfavorable learning environment. Therefore, it is important to limit the number of participants to up to eight.

Table 1: Sociodemographic data of participants from all three pilot evaluations

	**First pilot evaluation**	**Second pilot evaluation**		**Third pilot evaluation**
		Session 1	Session 2	
Gender *N* (%)				
Male	28 (90%)	9 (50%)	17 (40%)	3 (15%)
Female	3 (10%)	9 (50%)	26 (60%)	17 (85%)
Age (years)				
MD (SD)	28.2 (6.5)	32.5 (8.1)	35.1 (9.8)	36.5 (8.0)
Country of origin (n)[a]				
Afghanistan	-	8	15	2
Syria	28	4	13	2
Iraq	-	5	5	15
Pakistan	-	1	8	-
Other	3	-	1	1
Education* (in years)				
MD (SD)	-	8.2 (4.7)	8.0 (5.5)	-
Education in levels[b] (%)				
basic	19%	-	-	-
medium	23%	-	-	-
high-school diploma	29%	-	-	-
university	29%	-	-	-
Time spent in Germany[b] (in months)				
MD/modus	2	34.6	33.9	36-48[c]
Range	0.25-3	-	-	-

Note. [a] one missing value; [b] Education and time spent in Germany was not obtained in the same way in all studies because of the different contexts in which they took place; [c] categorical variable

Table 2: Mean scores of the third pilot evaluations and correlations to duration of stay

	MD	SD	r^a
Overall benefit	4.0	1.2	-.11
Knowledge on the development of mental disorders	3.8	1.0	.36
Knowledge on the mental health care offered in a country	3.1	0.9	.03
Knowledge on individual resilience and coping strategies	3.1	1.3	-.14
Group atmosphere	4.2	1.0	-.07
Concentration	4.3	0.9	-.04
Comprehensibility	4.4	0.6	-.24
Strengthening	3.8	1.1	-.23
Relief	3.8	1.2	-.10
Trust	4.1	1.2	-.02
Motivation for further participation	4.4	0.9	-.12
Group format	4.3	0.9	.11
Recommendation to others	4.3	0.9	.25

Note. 1=lowest to 5=highest/ best; [a] all non-significant (*p*s=.12 - .95).

References

Alhamad, A., & Al-Faris, E. A. (1998). The validation of the general health questionnaire (ghq-28) in a primary care setting in saudi arabia. *J Family Community Med*, *5*(1), 13-19. https://www.ncbi.nlm.nih.gov/pubmed/23008578

Bogdanski, C., Ghazal Makki, S., Hermann, A., Nater-Mewes, R., Diringer-Seither, A., & Stark, R. (2019). Evaluation des „Gesundheits-Teegarten“ – eine psychoedukative Gesprächsgruppe mit Geflüchteten [Evaluation of the health Teagarden - a psychoeducative discussion group with refugees]. *Gesundheitswesen*, *81*(03), 265. https://doi.org/10.1055/s-0039-1679354

Connor, K. M., & Davidson, J. R. (2003). Development of a new resilience scale: the Connor-Davidson Resilience Scale (CD-RISC). *Depress Anxiety*, *18*(2), 76-82. https://doi.org/10.1002/da.10113

Demir, S., Reich, H., & Mewes, R. (2016). Psychologische Erstbetreuung für Asylsuchende. Entwicklung und erste Erfahrungen mit einer Gruppenpsychoedukation für Geflüchtete [Psychological first aid for asylum seekers: Development and evaluation of psychoeducation for refugees]. *Psychotherapeutenjournal*, *2/2016*, 124-131.

Goldberg, D. (1978). *Manual of the General Health Questionnaire*. NFER-Nelson.

Mackenzie, C. S., Knox, V. J., Gekoski, W. L., & Macaulay, H. L. (2004). An adaptation and extension of the attitudes toward seeking professional psychological help scale. *Journal of Applied Social Psychology*, *34*(11), 2410-2435. https://doi.org/10.1111/j.1559-1816.2004.tb01984.x

Ormel, J., Koeter, M. W., van den Brink, W., & Giel, R. (1989). Concurrent validity of GHQ-28 and PSE as measures of change. *Psychol Med*, *19*(4), 1007-1013. https://www.ncbi.nlm.nih.gov/pubmed/2594877
